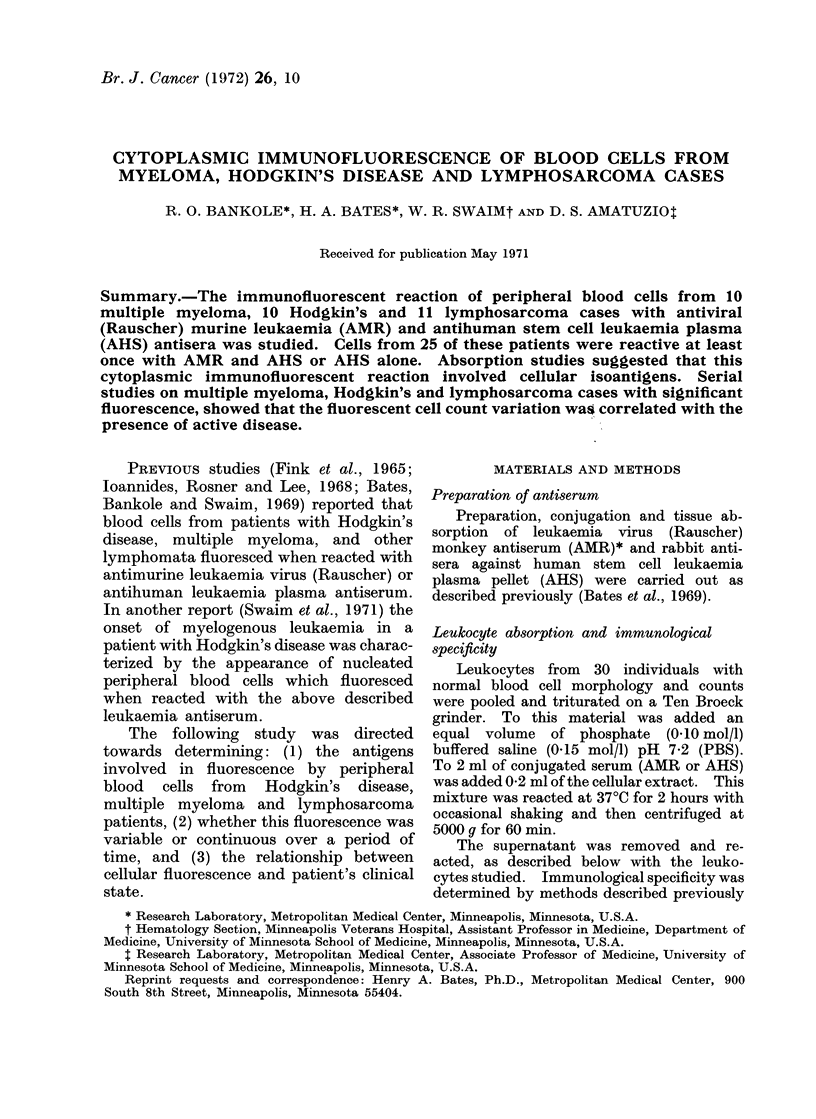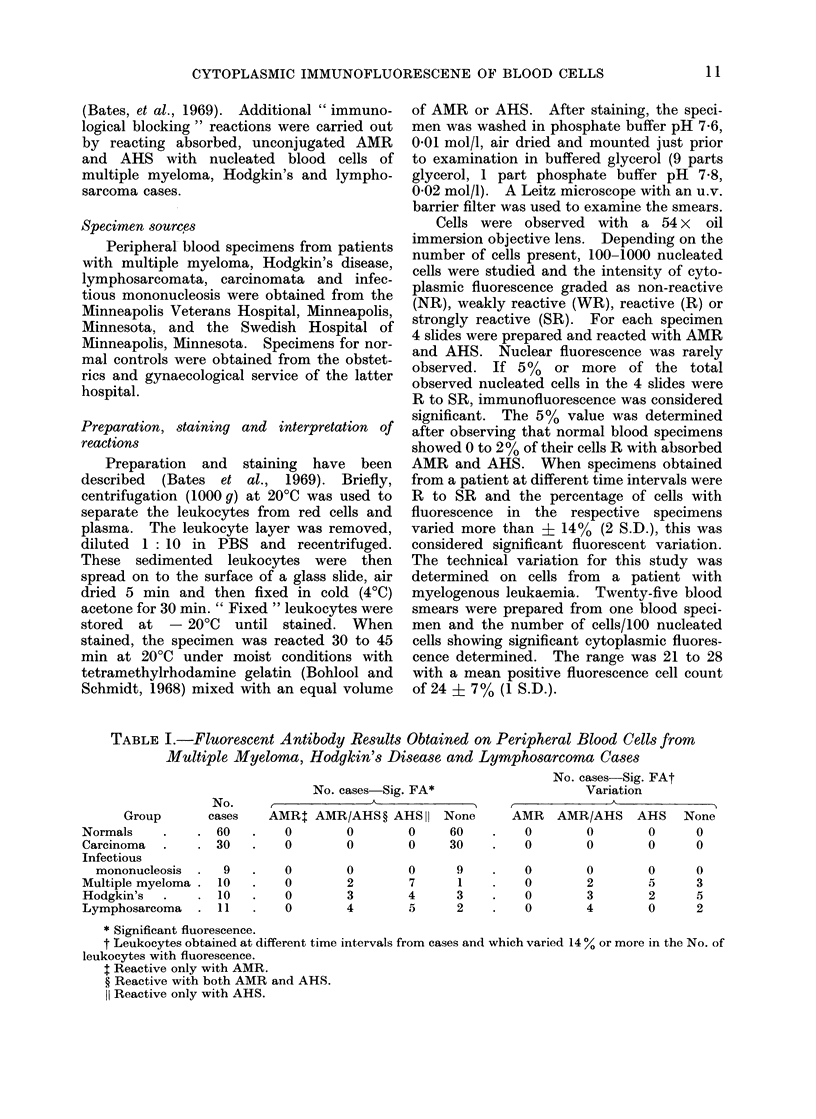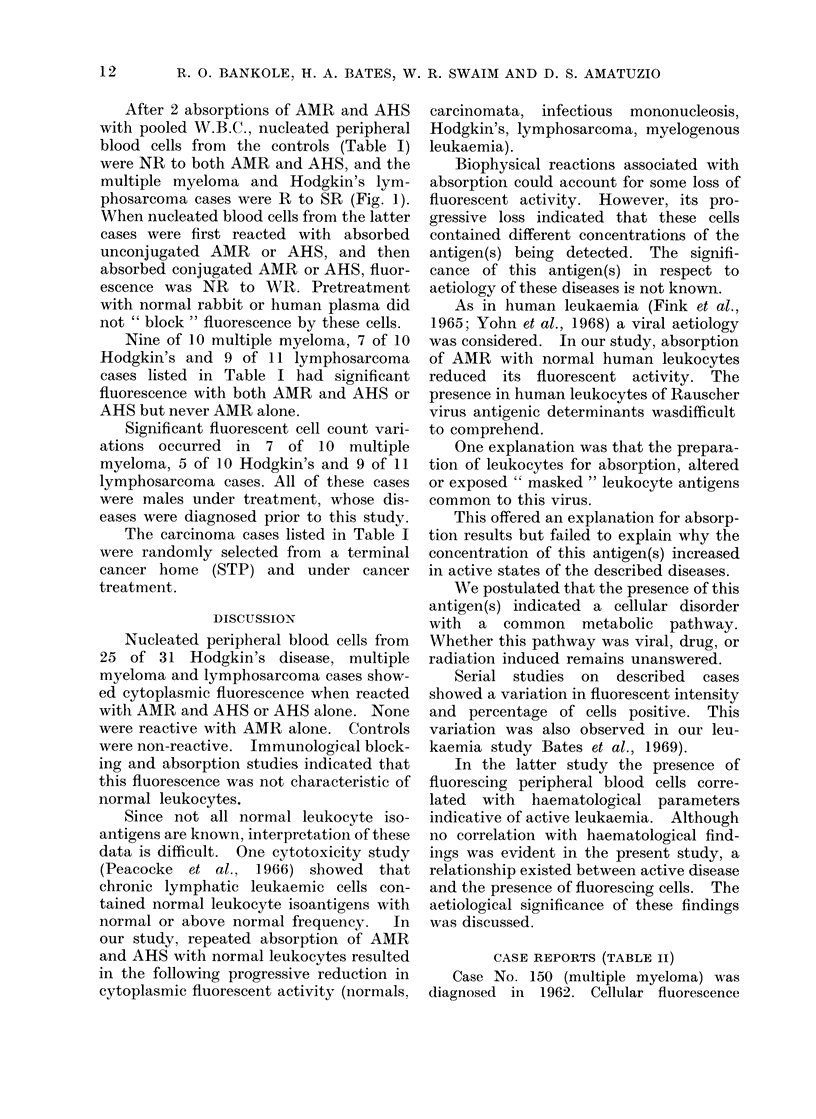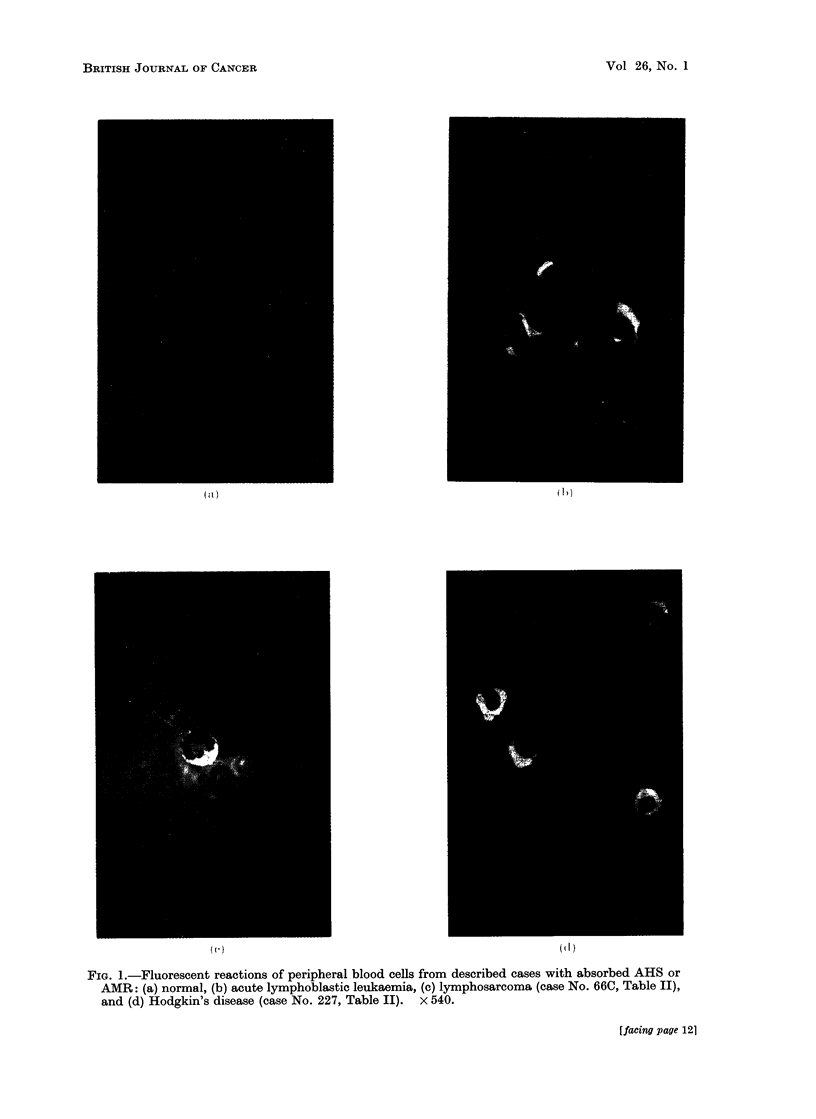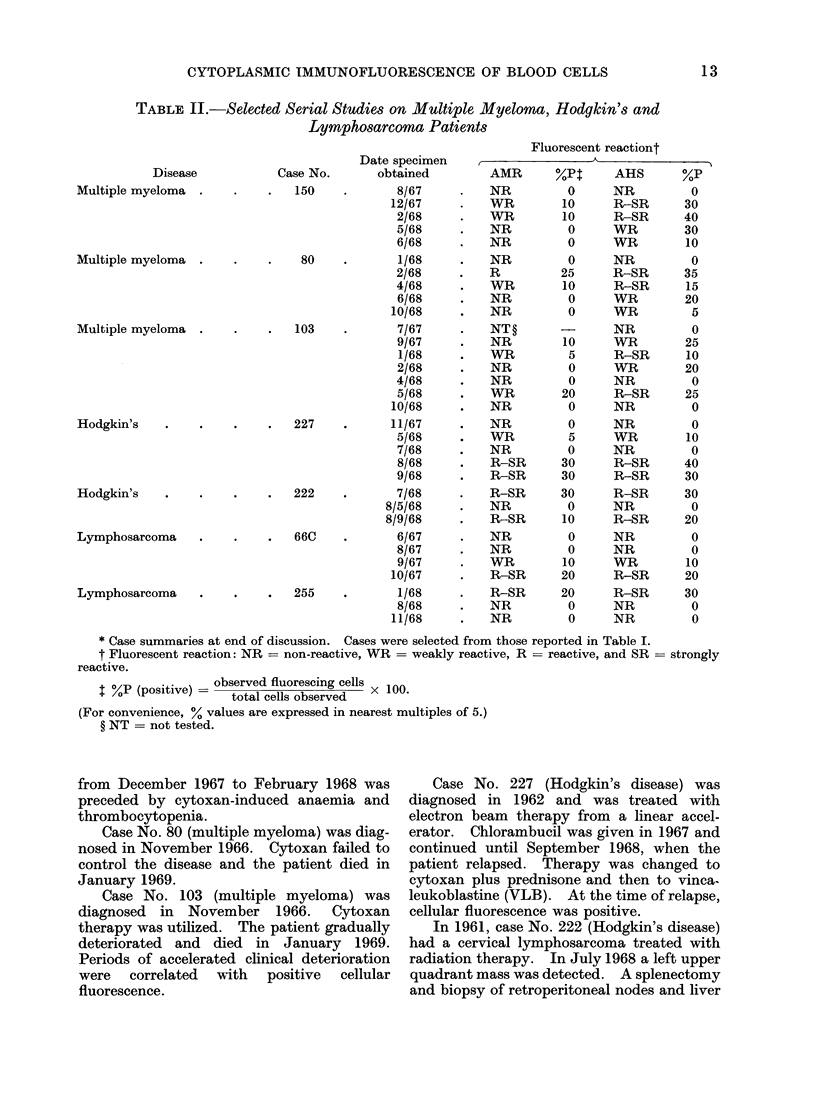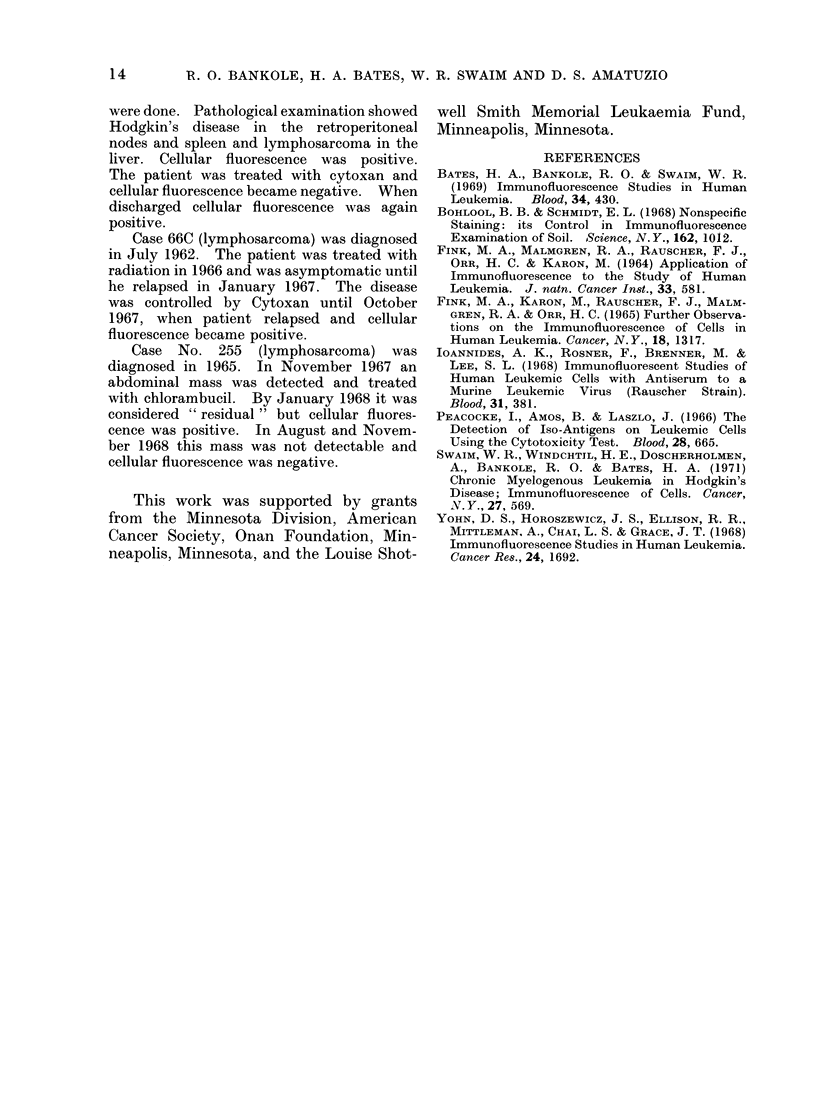# Cytoplasmic Immunofluorescence of Blood Cells from Myeloma, Hodgkin's Disease and Lymphosarcoma Cases

**DOI:** 10.1038/bjc.1972.4

**Published:** 1972-02

**Authors:** R. O. Bankole, H. A. Bates, W. R. Swaim, D. S. Amatuzio

## Abstract

**Images:**


*Br. J. Cancer* (1972) **26**, 10

### CYTOPLASMIC IMMUNOFLUORESCENCE OF BLOOD CELLS FROM MYELOMA, HODGKIN'S DISEASE AND LYMPHOSARCOMA CASES

R. O. BANKOLE*, H. A. BATES*, W. R. SWAIM† AND D. S. AMATUZIO‡

Received for publication May 1971

**Summary.—The immunofluorescent reaction of peripheral blood cells from 10 multiple myeloma, 10 Hodgkin's and 11 lymphosarcoma cases with antiviral (Rauscher) murine leukaemia (AMR) and antihuman stem cell leukaemia plasma (AHS) antisera was studied. Cells from 25 of these patients were reactive at least once with AMR and AHS or AHS alone. Absorption studies suggested that this cytoplasmic immunofluorescent reaction involved cellular isoantigens. Serial studies on multiple myeloma, Hodgkin's and lymphosarcoma cases with significant fluorescence, showed that the fluorescent cell count variation was correlated with the presence of active disease.**

PREVIOUS studies (Fink *et al.*, 1965; Ioannides, Rosner and Lee, 1968; Bates, Bankole and Swaim, 1969) reported that blood cells from patients with Hodgkin's disease, multiple myeloma, and other lymphomata fluoresced when reacted with antimurine leukaemia virus (Rauscher) or antihuman leukaemia plasma antiserum. In another report (Swaim *et al.*, 1971) the onset of myelogenous leukaemia in a patient with Hodgkin's disease was characterized by the appearance of nucleated peripheral blood cells which fluoresced when reacted with the above described leukaemia antiserum.

The following study was directed towards determining: (1) the antigens involved in fluorescence by peripheral blood cells from Hodgkin's disease, multiple myeloma and lymphosarcoma patients, (2) whether this fluorescence was variable or continuous over a period of time, and (3) the relationship between cellular fluorescence and patient's clinical state.

### MATERIALS AND METHODS

### *Preparation of antiserum*

Preparation, conjugation and tissue absorption of leukaemia virus (Rauscher) monkey antiserum (AMR)* and rabbit antisera against human stem cell leukaemia plasma pellet (AHS) were carried out as described previously (Bates *et al.*, 1969).

### *Leukocyte absorption and immunological specificity*

Leukocytes from 30 individuals with normal blood cell morphology and counts were pooled and triturated on a Ten Broeck grinder. To this material was added an equal volume of phosphate (0·10 mol/l) buffered saline (0·15 mol/l) pH 7·2 (PBS). To 2 ml of conjugated serum (AMR or AHS) was added 0·2 ml of the cellular extract. This mixture was reacted at 37°C for 2 hours with occasional shaking and then centrifuged at 5000 *g* for 60 min.

The supernatant was removed and reacted, as described below with the leukocytes studied. Immunological specificity was determined by methods described previously

* Research Laboratory, Metropolitan Medical Center, Minneapolis, Minnesota, U.S.A.
† Hematology Section, Minneapolis Veterans Hospital, Assistant Professor in Medicine, Department of Medicine, University of Minnesota School of Medicine, Minneapolis, Minnesota, U.S.A.
‡ Research Laboratory, Metropolitan Medical Center, Associate Professor of Medicine, University of Minnesota School of Medicine, Minneapolis, Minnesota, U.S.A.
Reprint requests and correspondence: Henry A. Bates, Ph.D., Metropolitan Medical Center, 900 South 8th Street, Minneapolis, Minnesota 55404.

(Bates, *et al.*, 1969). Additional " immunological blocking " reactions were carried out by reacting absorbed, unconjugated AMR and AHS with nucleated blood cells of multiple myeloma, Hodgkin's and lymphosarcoma cases.

*Specimen sources*

Peripheral blood specimens from patients with multiple myeloma, Hodgkin's disease, lymphosarcomata, carcinomata and infectious mononucleosis were obtained from the Minneapolis Veterans Hospital, Minneapolis, Minnesota, and the Swedish Hospital of Minneapolis, Minnesota. Specimens for normal controls were obtained from the obstetrics and gynaecological service of the latter hospital.

*Preparation, staining and interpretation of reactions*

Preparation and staining have been described (Bates *et al.*, 1969). Briefly, centrifugation (1000 *g*) at 20°C was used to separate the leukocytes from red cells and plasma. The leukocyte layer was removed, diluted 1 : 10 in PBS and recentrifuged. These sedimented leukocytes were then spread on to the surface of a glass slide, air dried 5 min and then fixed in cold (4°C) acetone for 30 min. " Fixed " leukocytes were stored at − 20°C until stained. When stained, the specimen was reacted 30 to 45 min at 20°C under moist conditions with tetramethylrhodamine gelatin (Bohlool and Schmidt, 1968) mixed with an equal volume of AMR or AHS. After staining, the specimen was washed in phosphate buffer pH 7·6, 0·01 mol/l, air dried and mounted just prior to examination in buffered glycerol (9 parts glycerol, 1 part phosphate buffer pH 7·8, 0·02 mol/l). A Leitz microscope with an u.v. barrier filter was used to examine the smears.

Cells were observed with a 54× oil immersion objective lens. Depending on the number of cells present, 100–1000 nucleated cells were studied and the intensity of cytoplasmic fluorescence graded as non-reactive (NR), weakly reactive (WR), reactive (R) or strongly reactive (SR). For each specimen 4 slides were prepared and reacted with AMR and AHS. Nuclear fluorescence was rarely observed. If 5% or more of the total observed nucleated cells in the 4 slides were R to SR, immunofluorescence was considered significant. The 5% value was determined after observing that normal blood specimens showed 0 to 2% of their cells R with absorbed AMR and AHS. When specimens obtained from a patient at different time intervals were R to SR and the percentage of cells with fluorescence in the respective specimens varied more than ± 14% (2 S.D.), this was considered significant fluorescent variation. The technical variation for this study was determined on cells from a patient with myelogenous leukaemia. Twenty-five blood smears were prepared from one blood specimen and the number of cells/100 nucleated cells showing significant cytoplasmic fluorescence determined. The range was 21 to 28 with a mean positive fluorescence cell count of 24 ± 7% (1 S.D.).

TABLE I.—*Fluorescent Antibody Results Obtained on Peripheral Blood Cells from Multiple Myeloma, Hodgkin's Disease and Lymphosarcoma Cases*

| Group | No. cases | No. cases—Sig. FA* | | | | No. cases—Sig. FA† Variation | | | |
|---|---|---|---|---|---|---|---|---|---|
| | | AMR‡ | AMR/AHS§ | AHS‖ | None | AMR | AMR/AHS | AHS | None |
| Normals | 60 | 0 | 0 | 0 | 60 | 0 | 0 | 0 | 0 |
| Carcinoma | 30 | 0 | 0 | 0 | 30 | 0 | 0 | 0 | 0 |
| Infectious mononucleosis | 9 | 0 | 0 | 0 | 9 | 0 | 0 | 0 | 0 |
| Multiple myeloma | 10 | 0 | 2 | 7 | 1 | 0 | 2 | 5 | 3 |
| Hodgkin's | 10 | 0 | 3 | 4 | 3 | 0 | 3 | 2 | 5 |
| Lymphosarcoma | 11 | 0 | 4 | 5 | 2 | 0 | 4 | 0 | 2 |

* Significant fluorescence.

† Leukocytes obtained at different time intervals from cases and which varied 14% or more in the No. of leukocytes with fluorescence.

‡ Reactive only with AMR.

§ Reactive with both AMR and AHS.

‖ Reactive only with AHS.

After 2 absorptions of AMR and AHS with pooled W.B.C., nucleated peripheral blood cells from the controls (Table I) were NR to both AMR and AHS, and the multiple myeloma and Hodgkin's lymphosarcoma cases were R to SR (Fig. 1). When nucleated blood cells from the latter cases were first reacted with absorbed unconjugated AMR or AHS, and then absorbed conjugated AMR or AHS, fluorescence was NR to WR. Pretreatment with normal rabbit or human plasma did not " block " fluorescence by these cells.

Nine of 10 multiple myeloma, 7 of 10 Hodgkin's and 9 of 11 lymphosarcoma cases listed in Table I had significant fluorescence with both AMR and AHS or AHS but never AMR alone.

Significant fluorescent cell count variations occurred in 7 of 10 multiple myeloma, 5 of 10 Hodgkin's and 9 of 11 lymphosarcoma cases. All of these cases were males under treatment, whose diseases were diagnosed prior to this study.

The carcinoma cases listed in Table I were randomly selected from a terminal cancer home (STP) and under cancer treatment.

### DISCUSSION

Nucleated peripheral blood cells from 25 of 31 Hodgkin's disease, multiple myeloma and lymphosarcoma cases showed cytoplasmic fluorescence when reacted with AMR and AHS or AHS alone. None were reactive with AMR alone. Controls were non-reactive. Immunological blocking and absorption studies indicated that this fluorescence was not characteristic of normal leukocytes.

Since not all normal leukocyte isoantigens are known, interpretation of these data is difficult. One cytotoxicity study (Peacocke *et al.*, 1966) showed that chronic lymphatic leukaemic cells contained normal leukocyte isoantigens with normal or above normal frequency. In our study, repeated absorption of AMR and AHS with normal leukocytes resulted in the following progressive reduction in cytoplasmic fluorescent activity (normals, carcinomata, infectious mononucleosis, Hodgkin's, lymphosarcoma, myelogenous leukaemia).

Biophysical reactions associated with absorption could account for some loss of fluorescent activity. However, its progressive loss indicated that these cells contained different concentrations of the antigen(s) being detected. The significance of this antigen(s) in respect to aetiology of these diseases is not known.

As in human leukaemia (Fink *et al.*, 1965; Yohn *et al.*, 1968) a viral aetiology was considered. In our study, absorption of AMR with normal human leukocytes reduced its fluorescent activity. The presence in human leukocytes of Rauscher virus antigenic determinants wasdifficult to comprehend.

One explanation was that the preparation of leukocytes for absorption, altered or exposed " masked " leukocyte antigens common to this virus.

This offered an explanation for absorption results but failed to explain why the concentration of this antigen(s) increased in active states of the described diseases.

We postulated that the presence of this antigen(s) indicated a cellular disorder with a common metabolic pathway. Whether this pathway was viral, drug, or radiation induced remains unanswered.

Serial studies on described cases showed a variation in fluorescent intensity and percentage of cells positive. This variation was also observed in our leukaemia study Bates *et al.*, 1969).

In the latter study the presence of fluorescing peripheral blood cells correlated with haematological parameters indicative of active leukaemia. Although no correlation with haematological findings was evident in the present study, a relationship existed between active disease and the presence of fluorescing cells. The aetiological significance of these findings was discussed.

### CASE REPORTS (TABLE II)

Case No. 150 (multiple myeloma) was diagnosed in 1962. Cellular fluorescence

FIG. 1.—Fluorescent reactions of peripheral blood cells from described cases with absorbed AHS or AMR: (a) normal, (b) acute lymphoblastic leukaemia, (c) lymphosarcoma (case No. 66C, Table II), and (d) Hodgkin's disease (case No. 227, Table II). ×540.

[*facing page* 12]

TABLE II.—*Selected Serial Studies on Multiple Myeloma, Hodgkin's and Lymphosarcoma Patients*

| Disease | Case No. | Date specimen obtained | Fluorescent reaction† AMR | %P‡ | AHS | %P |
|---|---|---|---|---|---|---|
| Multiple myeloma | 150 | 8/67 | NR | 0 | NR | 0 |
| | | 12/67 | WR | 10 | R–SR | 30 |
| | | 2/68 | WR | 10 | R–SR | 40 |
| | | 5/68 | NR | 0 | WR | 30 |
| | | 6/68 | NR | 0 | WR | 10 |
| Multiple myeloma | 80 | 1/68 | NR | 0 | NR | 0 |
| | | 2/68 | R | 25 | R–SR | 35 |
| | | 4/68 | WR | 10 | R–SR | 15 |
| | | 6/68 | NR | 0 | WR | 20 |
| | | 10/68 | NR | 0 | WR | 5 |
| Multiple myeloma | 103 | 7/67 | NT§ | — | NR | 0 |
| | | 9/67 | NR | 10 | WR | 25 |
| | | 1/68 | WR | 5 | R–SR | 10 |
| | | 2/68 | NR | 0 | WR | 20 |
| | | 4/68 | NR | 0 | NR | 0 |
| | | 5/68 | WR | 20 | R–SR | 25 |
| | | 10/68 | NR | 0 | NR | 0 |
| Hodgkin's | 227 | 11/67 | NR | 0 | NR | 0 |
| | | 5/68 | WR | 5 | WR | 10 |
| | | 7/68 | NR | 0 | NR | 0 |
| | | 8/68 | R–SR | 30 | R–SR | 40 |
| | | 9/68 | R–SR | 30 | R–SR | 30 |
| Hodgkin's | 222 | 7/68 | R–SR | 30 | R–SR | 30 |
| | | 8/5/68 | NR | 0 | NR | 0 |
| | | 8/9/68 | R–SR | 10 | R–SR | 20 |
| Lymphosarcoma | 66C | 6/67 | NR | 0 | NR | 0 |
| | | 8/67 | NR | 0 | NR | 0 |
| | | 9/67 | WR | 10 | WR | 10 |
| | | 10/67 | R–SR | 20 | R–SR | 20 |
| Lymphosarcoma | 255 | 1/68 | R–SR | 20 | R–SR | 30 |
| | | 8/68 | NR | 0 | NR | 0 |
| | | 11/68 | NR | 0 | NR | 0 |

* Case summaries at end of discussion. Cases were selected from those reported in Table I.
† Fluorescent reaction: NR = non-reactive, WR = weakly reactive, R = reactive, and SR = strongly reactive.

‡ $\%P \text{ (positive)} = \frac{\text{observed fluorescing cells}}{\text{total cells observed}} \times 100.$

(For convenience, % values are expressed in nearest multiples of 5.)
§ NT = not tested.

from December 1967 to February 1968 was preceded by cytoxan-induced anaemia and thrombocytopenia.

Case No. 80 (multiple myeloma) was diagnosed in November 1966. Cytoxan failed to control the disease and the patient died in January 1969.

Case No. 103 (multiple myeloma) was diagnosed in November 1966. Cytoxan therapy was utilized. The patient gradually deteriorated and died in January 1969. Periods of accelerated clinical deterioration were correlated with positive cellular fluorescence.

Case No. 227 (Hodgkin's disease) was diagnosed in 1962 and was treated with electron beam therapy from a linear accelerator. Chlorambucil was given in 1967 and continued until September 1968, when the patient relapsed. Therapy was changed to cytoxan plus prednisone and then to vincaleukoblastine (VLB). At the time of relapse, cellular fluorescence was positive.

In 1961, case No. 222 (Hodgkin's disease) had a cervical lymphosarcoma treated with radiation therapy. In July 1968 a left upper quadrant mass was detected. A splenectomy and biopsy of retroperitoneal nodes and liver were done. Pathological examination showed Hodgkin's disease in the retroperitoneal nodes and spleen and lymphosarcoma in the liver. Cellular fluorescence was positive. The patient was treated with cytoxan and cellular fluorescence became negative. When discharged cellular fluorescence was again positive.

Case 66C (lymphosarcoma) was diagnosed in July 1962. The patient was treated with radiation in 1966 and was asymptomatic until he relapsed in January 1967. The disease was controlled by Cytoxan until October 1967, when patient relapsed and cellular fluorescence became positive.

Case No. 255 (lymphosarcoma) was diagnosed in 1965. In November 1967 an abdominal mass was detected and treated with chlorambucil. By January 1968 it was considered " residual " but cellular fluorescence was positive. In August and November 1968 this mass was not detectable and cellular fluorescence was negative.

This work was supported by grants from the Minnesota Division, American Cancer Society, Onan Foundation, Minneapolis, Minnesota, and the Louise Shotwell Smith Memorial Leukaemia Fund, Minneapolis, Minnesota.

### REFERENCES

Bates, H. A., Bankole, R. O. & Swaim, W. R. (1969) Immunofluorescence Studies in Human Leukemia. *Blood*, **34**, 430.

Bohlool, B. B. & Schmidt, E. L. (1968) Nonspecific Staining: its Control in Immunofluorescence Examination of Soil. *Science, N.Y.*, **162**, 1012.

Fink, M. A., Malmgren, R. A., Rauscher, F. J., Orr, H. C. & Karon, M. (1964) Application of Immunofluorescence to the Study of Human Leukemia. *J. natn. Cancer Inst.*, **33**, 581.

Fink, M. A., Karon, M., Rauscher, F. J., Malmgren, R. A. & Orr, H. C. (1965) Further Observations on the Immunofluorescence of Cells in Human Leukemia. *Cancer, N.Y.*, **18**, 1317.

Ioannides, A. K., Rosner, F., Brenner, M. & Lee, S. L. (1968) Immunofluorescent Studies of Human Leukemic Cells with Antiserum to a Murine Leukemic Virus (Rauscher Strain). *Blood*, **31**, 381.

Peacocke, I., Amos, B. & Laszlo, J. (1966) The Detection of Iso-Antigens on Leukemic Cells Using the Cytotoxicity Test. *Blood*, **28**, 665.

Swaim, W. R., Windchtil, H. E., Doscherholmen, A., Bankole, R. O. & Bates, H. A. (1971) Chronic Myelogenous Leukemia in Hodgkin's Disease; Immunofluorescence of Cells. *Cancer, N.Y.*, **27**, 569.

Yohn, D. S., Horoszewicz, J. S., Ellison, R. R., Mittleman, A., Chai, L. S. & Grace, J. T. (1968) Immunofluorescence Studies in Human Leukemia. *Cancer Res.*, **24**, 1692.